# Development of a novel denture care agent with highly active enzyme, arazyme

**DOI:** 10.1186/s12903-021-01733-7

**Published:** 2021-07-22

**Authors:** Jong-Hoon Kim, Ha-Neul Lee, Seong-Kyeong Bae, Dong-Ha Shin, Bon-Hwan Ku, Ho-Yong Park, Tae-Sook Jeong

**Affiliations:** 1 Industrial Biomaterials Research Center, Korea Research Institute of Bioscience and Biotechnology, Daejeon, 34141 Republic of Korea; 2 Insect Biotech Co. Ltd., Daejeon, 34054 Republic of Korea

**Keywords:** Denture hygiene, Denture cleansers, Protease, Antimicrobial activity, Arazyme

## Abstract

**Background:**

The importance of efficient denture deposit removal and oral hygiene has been further underscored by the continuous increase of denture wearers. Denture hygiene management has also become an important aspect associated with denture-induced stomatitis. This study aims to evaluate the denture cleaning effect of arazyme, the metalloprotease produced from the *Serratia proteamaculans* HY-3. We performed growth inhibition tests against oral opportunistic pathogens to be used as a potential oral health care agent.

**Methods:**

The proteolytic activities of arazyme was evaluated over broad ranges of temperature, pH, and denture components compared to those of subtilisin in commercially available denture cleansers. The washing effects of arazyme were also measured by using homogeneously soiled EMPA 105 cottons. To investigate the denture cleaning capability of arazyme, artificially contaminated dentures were treated with arazyme, subtilisin (Everlase 6.0T), and Polident®, respectively. The growth kinetics of *Candida albicans*, *Enterococcus faecalis*, *Staphylococcus epidermis*, and *Streptococcus mutans* were evaluated in the presence of different concentrations of arazyme to estimate the prevention effects of arazyme against major oral opportunistic pathogens.

**Results:**

Arazyme showed strong proteolytic activities over wide temperature and pH ranges compared with the serine protease of the subtilisin family. Arazyme demonstrated efficient removal and decomposition of artificially contaminated dentures and showed explicit washing effects against soiled cottons. Moreover arazyme inhibited the growth of oral opportunistic pathogens, including *C. albicans*, *E. faecalis*, *S. epidermis*, and *S. mutans*, with more than 80% inhibition against *C*. *albicans*, the major cause of denture stomatitis, with 250 mg/mL arazyme.

**Conclusions:**

Arazyme shows promise as a biological oral health care agent with effective cleaning and antimicrobial activities and is a potential source for developing novel denture care agents.

## Background

The importance of the efficient removal of denture deposits and hygiene management of dentures has been further underscored by the continuous increase in adult average life expectancy. According to the World Health Organization, around 30% of the global population aged 65–74 years is edentulous, many of whom replace their lost teeth with dentures.

Denture stomatitis is an inflammatory reaction of the palatal mucosa, resulting in removable dentures characterized by different erythema levels. Denture stomatitis is one of the most common problems for elderly people who wear partial or complete dentures, and the disease affects 30–77.5% of denture wearers [1–4]. Although various factors affect the onset and severity of the disease, the most common causative factors are poor denture fit, increased age of dentures and wearers, *Candida* spp. infection, and poor denture hygiene [2, 5, 6]. In addition, various microbes proliferate through the deposits and plaque attached to the denture surface, causing odor and oral diseases, and a connection with systemic diseases, such as pneumonia and diabetes, has also been reported [7–11]. Therefore, denture plaque control has become an important aspect in denture-induced stomatitis associated with opportunistic microbial infections [12]. They are mechanical methods using brush or ultrasonic agitation, and chemical methods using immersion detergents, disinfectants and enzymes [13, 14]. An ideal strategy for plaque control is the combination of both mechanical and chemical methods [15]. Various denture cleansers that contain a primary component of enzymes, alkaline peroxides, or acids are available to remove the residual biofilm attached to denture surfaces [13, 16–18]. For denture cleansers, numerous types of inorganic (oxone, sodium bicarbonate, and sodium percarbonate) and organic (TAED, citric acid, and enzyme) components are used in formulations.

Proteolytic enzymes, which degrade proteins by cleaving peptide bonds, were the first enzymes to be included in detergents and remain the most commonly used primary component in detergents in general. Several detergent-stable proteases have also been studied [19–22]. The most widely used protease is subtilisin, which is derived from *Bacillus* species. Subtilisin is a non-specific serine protease that provides the preferred cleavage on the carboxyl side of hydrophobic amino acid residues but can hydrolyze most peptide links [23]. As the proteolytic enzymes currently included in denture cleansers are limited to those in the subtilisin family, substantial effort has been invested into developing a novel proteolytic enzyme or modifying existing subtilisin with tolerance to the detergent components [23–25].

Previously, we isolated *Serratia proteamaculans* HY-3, a symbiotic bacterium of the spider *Nephila clavata*, which excretes a 51.5 kDa metalloprotease, arazyme [26]. Purified arazyme showed high relative proteolytic activities over wide temperature and pH ranges [27, 28]. Therefore, the present study aimed to evaluate the efficacy of arazyme to determine its possible applications as a denture cleanser for oral health care.

## Methods

### Evaluation of proteolytic activities

The arazyme, which was evaluated for its denture cleanser properties, was provided by InsectBiotech Co., Ltd (Daejeon, Republic of Korea). The proteolytic activity of arazyme was determined using the method described by Mazorra-Manzano et al*.* [29] with minor modifications. Serine protease (Everlase 6.0T; Novozymes, Bagsværd, Denmark) of the subtilisin (EC 3.4.21.62) family contained in Polident®, a denture cleanser, was used as a positive control for the comparative evaluation of the proteolytic activity. Briefly, 500 μL of 0.6% (w/v) casein solution in 0.05 M disodium phosphate buffer (pH 7.5) was added to 100 μL of the samples and incubated at 37 °C for 10 min. After incubation, 500 μL of 1.8% (w/v) trichloroacetic acid was added to each tube, and the mixture was incubated at 37 °C for 30 min to stop the reaction. The solution was then centrifuged at 10,000 × *g* and 4 °C for 10 min. An aliquot of 200 μL of the supernatant was added to 500 μL of 0.55 M sodium carbonate and 100 μL of 0.5 N Folin-Ciocalteu phenol reagent, followed by incubation at 37 °C for 30 min. After incubation, the absorbance of produced L-tyrosine was measured using a spectrophotometer (DU 730® Life Science UV/Vis spectrophotometer; Beckman Coulter, Brea, CA, USA) at 660 nm.

The effects of temperature (20–100 °C with pH 7.5) and pH (5.0–10.0 at 37 °C) on the proteolytic activity of the arazyme were also investigated. To investigate the influence of detergent components on proteolytic activity, various detergent components (surfactant: sodium dodecyl sulfate; bleaching agents: sodium bicarbonate, sodium perborate, sodium percarbonate, and tetraacetylethylenediamine; pH adjustment: citric acid; non-chlorine oxidizer: potassium peroxymonosulfate; anti-redeposition agent; polyethylene glycol, Table 1) were added to the standard assay reactions as described above.

**Table 1:** Stability of arazyme in the presence of various detergent components

Detergent components	Concentration (v/v, %)	Residual activity (%)	
		Arazyme	Everlase 6.0T
None	–	100	100
Surfactant			
SDS	25	107	104
Bleaching agents			
Sodium bicarbonate	25	110	103
Sodium perborate	25	97	110
Sodium percarbonate	25	105	99
TAED	25	92	90
pH adjustment			
Citric acid	2	98	91
Non-chlorine oxidizer			
Potassium peroxymonosulfate	25	103	98
Anti-redeposition agent			
PEG	2	100	100

*SDS* sodium dodecyl sulfate, *TAED* tetraacetylethylenediamine, *PEG* polyethylene glycol

Data are presented as means (*n* = 3)

### Evaluation of washing performance on EMPA 105 cotton

The application of arazyme as a detergent was evaluated on EMPA 105 cotton (4.5 × 7.5 cm) homogeneously soiled with blood pig, cocoa, and red wine (EMPA-Testfabrics, West Pittston, PA, USA). Each soiled cotton sample was washed by immersion in 200 mL of water each containing arazyme (220 μg) and Everlase 6.0T (13.75 mg), respectively, with a corresponding active enzyme unit for 12 h at 25 °C. The active unit of enzyme used in the test was based on the content added per tablet of Polident®. One tablet of Polident® was used as a positive control. The color parameters, including color depth (K/S value), brightness (L*), red-green (a*), and yellow-blue (b*), were determined using a spectrophotometer (ColorTouch® II; Technidyne Co., New Albany, IN, USA). The washing tests were performed in triplicate under the same conditions.

### Denture cleaning capacity

To investigate the denture cleaning capability of arazyme, artificially contaminated dentures were treated with arazyme and positive control (Everlase 6.0T and Polident® 3–minute denture cleanser). The dentures were immersed in 10% whole milk powder (SeoulMilk Co., Seoul, Korea) a composite substrate with carbohydrates, proteins, and fats and the process of drying for 10 min was repeated five times. For working solutions, arazyme (220 μg and 13.75 mg), Everlase 6.0T (13.75 mg), and one tablet of Polident® were dissolved in 200 mL of tap water, and the solution was handled immediately. The contaminated dentures were soaked in each solution at 25 °C for 1 h. Cleaning solutions were used only once, and fresh materials were used for each replicate experiment. The negative control group was soaked only in water. The soiled dentures were stained with 0.05% Coomassie Brilliant Blue R-250 (Bio-Rad, Hercules, CA, USA) reagent and subsequently rinsed in tap water to remove any unbound dye. The treated and untreated dentures were compared using the MetaMorph Imaging System (Meta Imaging Software, Synnyvale, CA, USA) to evaluate the efficacy of arazyme treatment. The experiments were performed in triplicate under the same conditions.

### Antimicrobial assay

Three gram-positive bacteria, *Enterococcus faecalis* KACC 11859, *Staphylococcus epidermis* KACC 13234, *Streptococcus mutans* KACC 16833, and the yeast *Candida albicans* KACC 30071 from Korean Agricultural Culture Collection (KACC) were used to evaluate the antimicrobial activity of arazyme in the present study. The inhibition of bacterial growth by arazyme was determined using the method previously described [30] with minor modifications.

The overnight-cultured cells of *E. faecalis*, *S. epidermis*, and *S. mutans* grown in brain heart infusion medium (0.77% calf brain, 0.98% beef heart, 1% proteose peptone, 0.2% dextrose, 0.5% sodium chloride, and 0.25% disodium phosphate) and *C. albicans* grown in yeast malt medium (0.3% yeast extract, 0.3% malt extract, 0.5% peptone, and 1% dextrose) were seeded into each well of a 96-well plate with a final density of 2.0 × 10^6^ CFU/mL. Arazyme was then added at a final concentration of 10–250 mg/mL. The cells were subsequently grown in the 96-well plate at 30 °C for 24 h. The growth curve was confirmed by measuring absorbance at 600 nm using a spectrophotometer for one day at 3 h intervals. Growth curves are represented as the time dependence of the optical density at 600 nm.

### Statistical analysis

One-way analysis of variance was performed using SPSS software (version 24, SPSS, Inc., Chicago, IL, USA). The mean values were compared using Scheffé’s method, and a *p* value < 0.05 was considered significant.

## Results

### Proteolytic activity of arazyme

**Fig. 1:**
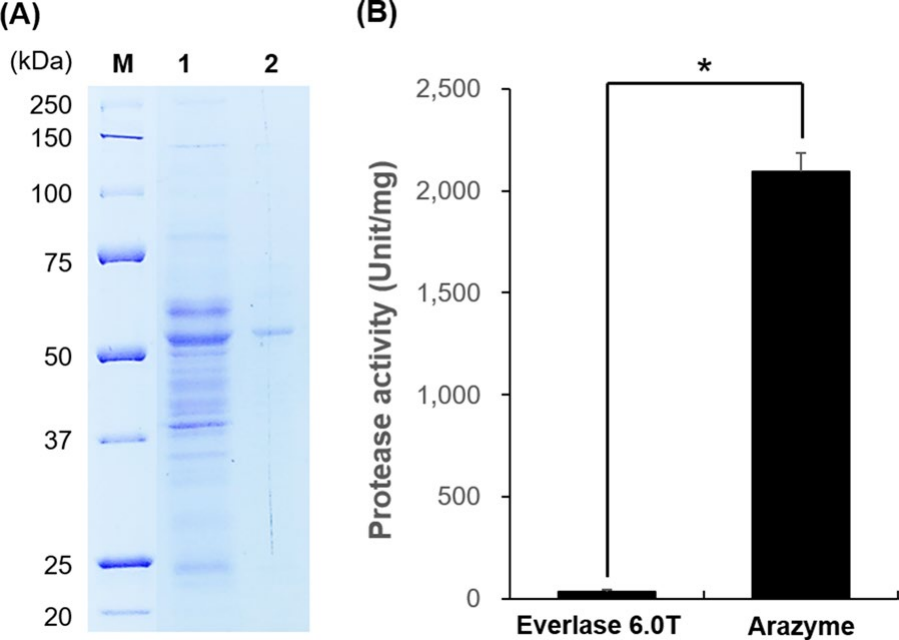
SDS-PAGE profiled **a** of purified arazyme from *S*. *proteamaculans* HY-3 and comparison of proteolytic activity with Everlase 6.0T used in denture cleanser (**b**). The purified enzyme was loaded on a 12% SDS polyacrylamide gel and stained with Coomassie brilliant blue. Lane M: molecular weight marker; Lane 1: extracellular fraction of *S*. *proteamaculans* HY-3; Lane 2: purified arazyme. Data are presented as means ± SD (*n* = 3). * Indicates by *P* < 0.05 versus the Everlase 6.0T

The purified arazyme showed relatively high proteolytic activity with 2094 ± 86 unit/mg. SDS-PAGE profile of the arazyme showed an intense and clear band with a molecular weight of 51.5 kDa (Fig. 1a). The positive control, Everlase 6.0T showed proteolytic activity of 33 ± 10 unit/mg which was about 63-fold lower than that of arazyme (Fig. 1b).

### Effects of arazyme on temperature, pH, and detergent components

**Fig. 2:**
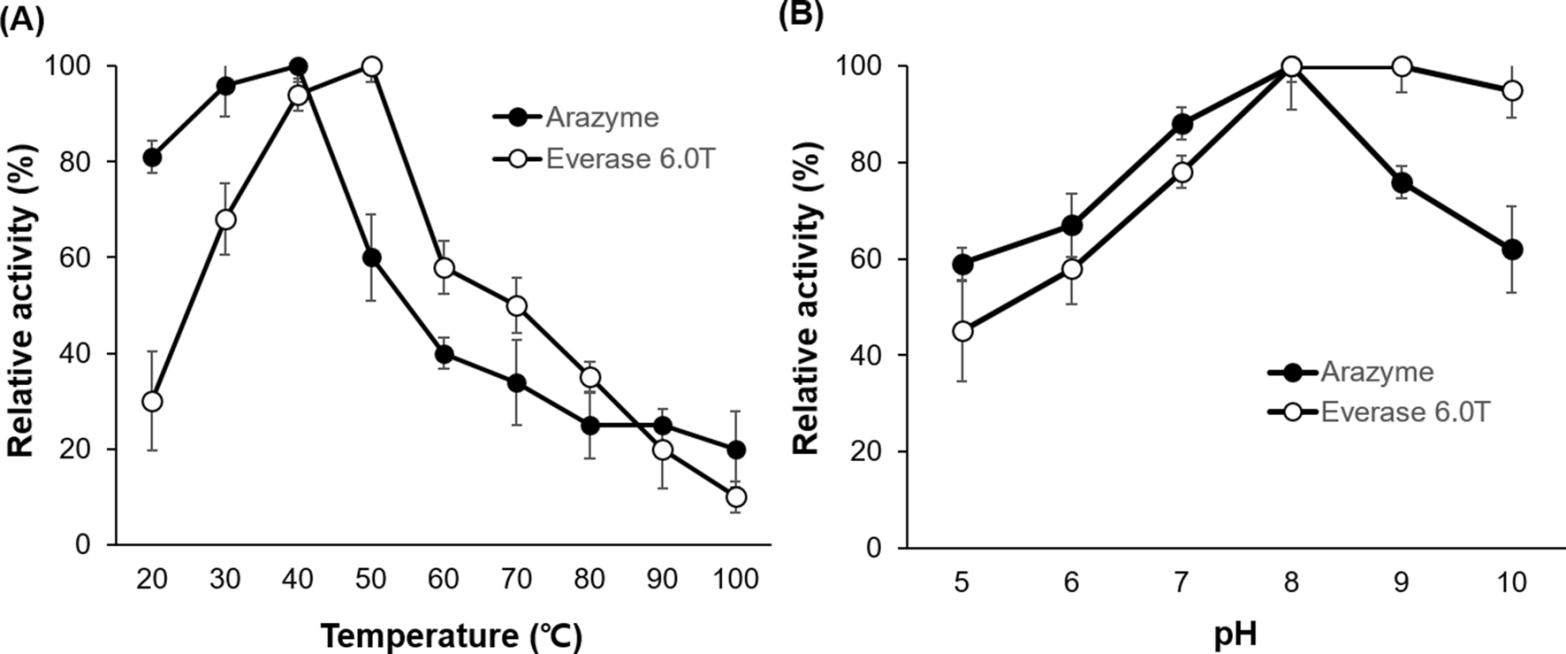
**a** Effect of temperature on the proteolytic activity. The temperature profiles of proteolytic activity treated with each protease were determined at temperature between 20 and 100 °C. **b** Effect of pH on the activity of arazyme and Everlase 6.0T. The proteolytic activities were assayed in the pH range of 5 to 10 using buffers of different pH values. Data are presented as means ± SD (*n* = 3)

The effect of temperature on proteolytic activity was determined over the range of 20–100 °C with pH 7.5. The activity changed with temperature in a manner typical of proteases for both arazyme and Everlase 6.0T (Fig. 2a). The temperature activity profile of arazyme showed that the optimal temperature (37 °C) was much lower than that of Everlase 6.0T (45 °C). The effect of pH on the casein hydrolysis was determined for the pH range of 5.0–10.0 at 37 °C (Fig. 2b). The arazyme and Everlase 6.0T showed a broader pH profile for proteolytic activity. Arazyme was highly active in the pH range of 5.0–10.0, with 80% of its maximum activity observed at an optimal pH of 8.0. The pH activity profile of Everlase 6.0T showed that the enzyme was highly active in the pH range of 8.0 to 10.0, with an optimal pH of 9.0. The effects of various detergent components on proteolytic activity are described in Table 1. The results showed that the aforementioned factors had no inhibitory effects on the proteolytic activity of arazyme or Everlase 6.0T.

### Effect of arazyme on soiled cottons

As shown in Table 2, a limited cleaning effect was observed with the enzyme only; however, following arazyme treatment, the color strength (K/S) value of arazyme of the blood, cocoa, and red wine-soiled cottons clearly decreased to 0.89, 2.42, and 0.51, respectively, as compared with those of the non-treated soiled cottons 1.20, 4.47, and 0.79, respectively. The treatment of soiled cottons with Everlase 6.0T showed a minor effect on K/S values than non-treated soiled cottons.

**Table 2:** Wash performance of arazyme, everlase 6.0T, and Polident® at 25 °C

EMPA cottons	Samples	L*	a*	b*	K/S value	Color
Blood	Water	58.95 ± 0.25^a^	8.56 ± 0.46^a^	20.35 ± 0.23^a^	1.20 ± 0.15^b^	
	Arazyme (220 μg)	51.87 ± 0.37^c^	3.74 ± 0.53^b^	17.73 ± 0.65^c^	0.89 ± 0.13^c^	
	Everlase 6.0T (13.75 mg)	55.83 ± 0.67^b^	8.84 ± 0.63^a^	19.67 ± 0.27^b^	1.50 ± 0.17^a^	
	Polident® (1 tablet)	50.73 ± 0.54^ cd^	2.29 ± 0.43^c^	15.34 ± 0.36^d^	0.46 ± 0.09^d^	
Cocoa	Water	62.05 ± 0.34^a^	4.44 ± 0.26^a^	18.12 ± 0.19^a^	4.47 ± 0.14^a^	
	Arazyme (220 μg)	59.68 ± 0.33^b^	1.35 ± 0.21^c^	15.75 ± 0.34^b^	2.42 ± 0.21^c^	
	Everlase 6.0T (13.75 mg)	60.13 ± 0.23^b^	3.84 ± 0.33^b^	18.02 ± 0.27^a^	3.89 ± 0.37^b^	
	Polident® (1 tablet)	58.89 ± 0.18^c^	3.36 ± 0.27^b^	13.48 ± 0.31^c^	0.57 ± 0.26^d^	
Red wine	Water	77.23 ± 0.35^a^	1.35 ± 0.12^a^	9.42 ± 0.42^a^	0.79 ± 0.11^a^	
	Arazyme (220 μg)	71.56 ± 0.77^d^	1.02 ± 0.17^b^	7.23 ± 0.38^b^	0.51 ± 0.12^c^	
	Everlase 6.0T (13.75 mg)	75.28 ± 0.46^b^	1.27 ± 0.09^a^	9.38 ± 0.47^a^	0.75 ± 0.15^b^	
	Polident® (1 tablet)	74.37 ± 0.14^c^	0.71 ± 0.13^c^	4.73 ± 0.26^c^	0.18 ± 0.14^d^	

L* brightness, a* red-green, b* yellow-blue, *K/S value*: color depth

^a^^−^^d^Mean ± SD (*n* = 3) within columns followed by the different letters indicate a significant difference by Scheffé’s test (*P* < 0.05). One tablet of Polident is 27,210 mg

### Denture cleaning effect of arazyme

**Fig. 3:**
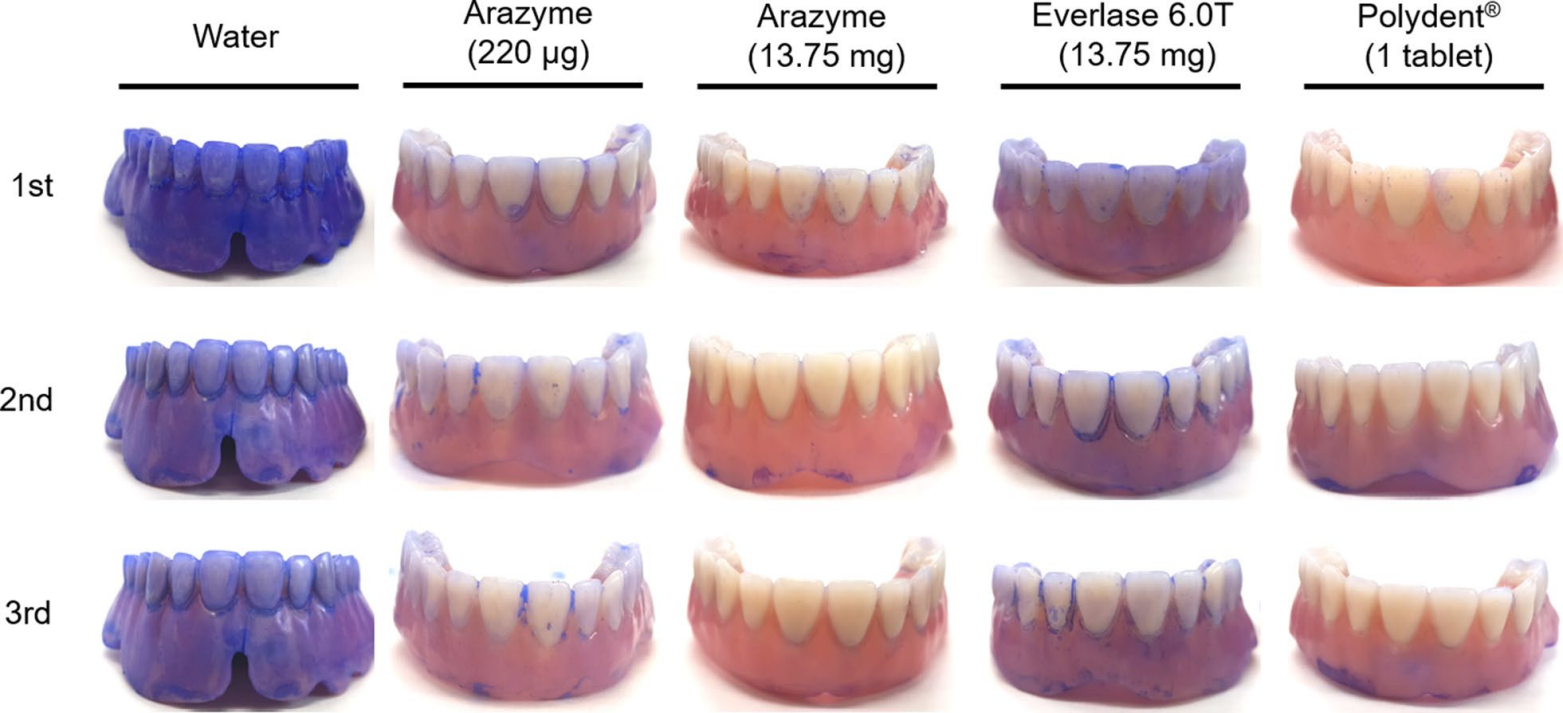
Evaluation of the cleaning abilities of arazyme. 220 μg and 13.75 mg of arazyme, 13.75 mg of Everlase 6.0T, and one tablet of Polident® were dissolved in 200 mL of tap water, respectively. The contaminated dentures were soaked in each solution at 25 °C for 1 h. Treated dentures were stained with a 0.05% Coomassie Brilliant Blue R-250 (Bio-Rad, USA) reagent and subsequently rinsed in tap water to remove any unbound dye. The experiments were performed in triplicate under the same conditions

To investigate the denture cleaning effect, arazyme and the positive controls (Everlase 6.0T and Polident®) were applied to each artificially contaminated denture (Fig. 3 and Table 3). The arazyme tests (220 μg and 13.75 mg) showed much lower normalized stain intensity when compared with the negative control group stained with 0.05% Coomassie Brilliant Blue R-250 reagent. In particular, 13.75 mg of arazyme displayed much lower staining densities than those of the 13.75 mg of Everlase 6.0T. Compared to Polident®, which contains Everlase 6.0T and various detergent components, arazyme had solely high denture cleaning ability. Besides, arazyme showed good cleaning performance even at room temperature, which is lower than the recommended use temperature of currently available denture cleansers such as Polident® or similar.

**Table 3:** Normalized staining intensity of artificially contaminated dentures after treatment

Samples	Treatment condition	Normalized intensity
Water	–	137.8 ± 4.8^a^
Arazyme	220 μg	115.0 ± 6.6^b^
	13.75 mg	96.4 ± 4.2^c^
Everlase 6.0T	13.75 mg	125.9 ± 5.2^b^
Polydent®	2710 mg (1 tablet)	90.4 ± 0.8^d^

^a^^−^^d^Mean ± SD (*n* = 3) within columns followed by the different letters indicate a significant difference by Scheffé’s test (*P* < 0.05)

### Effect of arazyme on microorganism growth

**Fig. 4:**
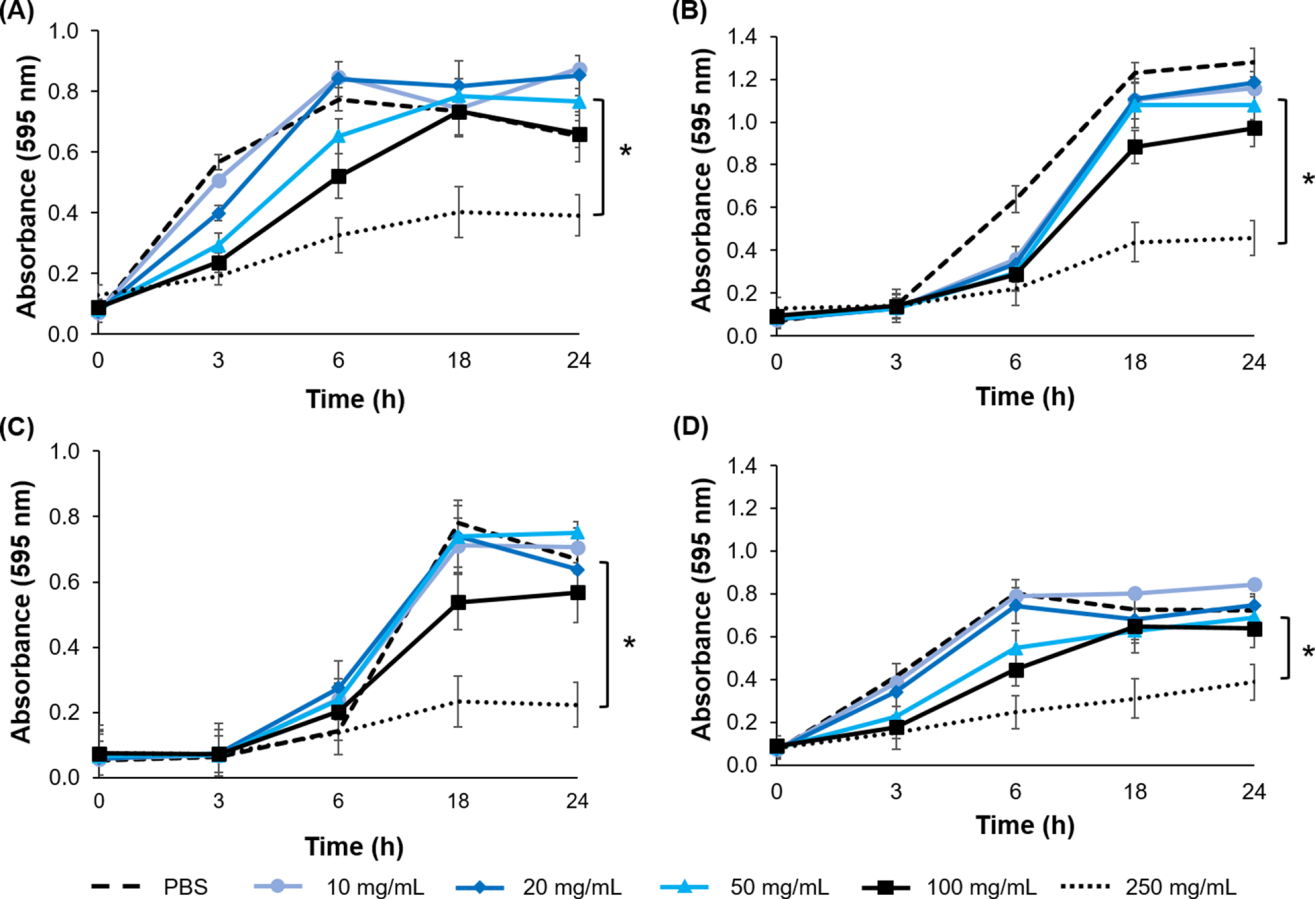
Inhibition growth curve of *E. faecalis* (**a**), *S. epidermis* (**b**), *C. albicans* (**c**), and *S. mutans* (**d**). To estimate antimicrobial activity, corresponding concentrations (10, 20, 50, 100, and 200 mg/mL) of arazyme were applied to each growth inhibition assay. The treated cells were incubated with shaking, and the optical density at 595 nm of each sample was measured every 3 h for 1 day. Data are presented as means ± SD (*n* = 3). Statistical significance between compared groups are indicated as * *P* < 0.05

The growth kinetics of *E. faecalis*, *S. epidermis*, *S. mutans*, and *C. albicans* in the presence of different concentrations of arazyme are shown in Fig. 4. Controls treated with phosphate-buffered saline for all microorganisms had typical growth curves with lag, logarithmic, and stationary phases. In contrast, all tested microorganism isolates were susceptible to arazyme at a concentration of 250 mg/mL. In addition, the growth of *C. albicans* was markedly inhibited when treated with arazyme. When the concentration of arazyme was 250 mg/mL, the growth of *C. albicans* was inhibited by over 80% (Fig. 4c).

## Discussion

Arazyme was characterized as maintaining high activities over broad temperature and pH ranges, efficiently hydrolyzing a broad range of substrates including albumin, keratin, and collagen [27, 31]. It is highly active over a broad pH range and is thus considered to play a role in aiding the digestion of spiders as spiders secrete powerful digestive enzymes that are injected into their captured prey to allow for their rapid decomposition.

Consistently, in this study, arazyme showed relatively high proteolytic activities over wide pH and temperature ranges. In addition, the proteolytic activities of arazyme were approximately 63-fold higher than those of Everlase 6.0T, which is a serine protease of the subtilisin family (EC 3.4.21.62). Enzymes are typically incorporated into detergent formulations at a low concentration (from 0.0005 to 0.5% active pure protein by weight) [19, 22, 32, 33]. Therefore, arazyme ability to hydrolyze casein at medium to high temperatures and over a relatively wide range of pH makes it an excellent complement to a potentially available protease of denture cleansers. The compatibility of the arazyme with various components of denture cleansers was also investigated. Arazyme showed excellent stability toward all of the commercial detergent components of denture cleansers tested, and the positive control Everlase 6.0T was also stable under the same conditions. Commercially available proteases such as subtilisin, alcalase, esparase, and savinase have been shown to exhibit great stability in the presence of detergents or surfactants [34], making them suitable as detergent additives. Taken together, these results demonstrated that the arazyme could be used as one of the main denture cleanser additives.

Several reports have demonstrated the usefulness of microbial-derived proteases in promoting the removal of blood, egg yolk, and chocolate stains from cotton cloth [35–38]. The present cleaning ability evaluation using soiled EMPA cotton confirmed that the arazyme itself could effectively remove various stains with blood, cocoa, and red wine. These results are consistent with previous reports on protease washing performance, which could efficiently remove stains by hydrolyzing large insoluble protein fragments firmly adhered to fabrics by synergizing with detergents [39, 40]. Furthermore, evaluating of the cleaning effect against artificially contaminated dentures showed that the contaminated deposit levels of arazyme-treated dentures were significantly low, demonstrating the same effect as the commercially available positive control Polident®. The effects of denture cleanser on acrylic resin surface roughness is important, because it can affect surface staining and biofilm accumulation. In previous studies, denture cleansers containing neutral peroxide with enzymes could effectively remove pellicles formed on the resin surface without altering the acrylic resin surface properties [41–43]. However, for an enzyme to be used as a constituent of a denture cleanser, it seems necessary to accurately measure the correlation between the immersion period and its influence on the acryl resin surface.

Denture stomatitis is a disease of fungal and bacterial origin. *C. albicans* is most often isolated from the oral cavities of these patients with denture stomatitis. This opportunistic pathogen can cause denture stomatitis and a wide spectrum of systemic diseases such as pneumonia and diabetes [1, 2, 44–46]. Arazyme exhibited antimicrobial activity of the four tested oral related opportunistic pathogens at a concentration of 250 mg/mL; in particular, *C. albicans* was one of the most susceptible pathogens tested, demonstrating the application prospects for arazyme to prevent denture stomatitis. The findings of this study demonstrate the limited sensitivity of this approach because of the concentration of the protease used; however, it still warrants further investigation to understand antimicrobial mechanisms of metalloproteases. Although arazyme showed denture cleansing and growth inhibition of some microbes, denture cleanser should be able to suppress biofilm accumulation without affecting the denture. Therefore, further studies such as antibiofilm evaluation for denture hygiene are needed to increase the practical applicability of these findings.

## Conclusion

The arazyme derived from *S. proteamaculans* HY-3 showed outstanding cleaning effects, along with high proteolytic activity under various conditions. These results suggest that arazyme is a promising source for the development of a denture cleanser in the course of exploring natural products with effectiveness and safety.

## Data Availability

The datasets used and/or analyzed during the current study are available from the corresponding author on reasonable request.

## References

[1] Arendorf T, Walker D (1987). Denture stomatitis: a review. J Oral Rehabil.

[2] Gendreau L, Loewy ZG (2011). Epidemiology and etiology of denture stomatitis. J Prosthodont.

[3] Puryer J (2016). Denure stomatitis: a clinical update. Dent Update.

[4] Johnson CC, Yu A, Lee H, Fidel PL, Noverr MC (2012). Development of a contemporary animal model of *Candida albicans*-associated denture stomatitis using a novel intraoral denture system. Infect Immun.

[5] Gasparoto TH, Vieira NA, Porto VC, Campanelli AP, Lara VS (2009). Ageing exacerbates damage of systemic and salivary neutrophils from patients presenting *Candida*-related denture stomatitis. Immun Ageing.

[6] Skupien JA, Valentini F, Boscato N, Pereira-Cenci T (2013). Prevention and treatment of *Candida* colonization on denture liners: a systematic review. J Prosthet Dent.

[7] Marsh P, Percival R (2006). The oral microflora—friend or foe? Can we decide?. Int Dent J.

[8] Coco B, Bagg J, Cross L, Jose A, Cross J, Ramage G (2008). Mixed *Candida albicans* and *Candida glabrata* populations associated with the pathogenesis of denture stomatitis. Oral Microbiol Immunol.

[9] Scannapieco FA, Shay K (2014). Oral health disparities in older adults: oral bacteria, inflammation, and aspiration pneumonia. Dent Clin N Am.

[10] Hannah VE, O'Donnell L, Robertson D, Ramage G (2017). Denture stomatitis: causes, cures and prevention. Prim Dent J.

[11] Kusama T, Aida J, Yamamoto T, Kondo K, Osaka K (2019). Infrequent denture cleaning increased the risk of pneumonia among community-dwelling older adults: a population-based cross-sectional study. Sci Rep.

[12] Douglas LJ (2003). *Candida* biofilms and their role in infection. Trends Microbiol.

[13] Keng S-B, Lim M (1996). Denture plaque distribution and the effectiveness of a perborate-containing denture cleanser. Quintessence Int (Berl).

[14] Ödman PA (1992). The effectiveness of an enzyme-containing denture cleanser. Quintessence Int (Berl).

[15] de Sousa Porta SR, de Lucena-Ferreira SC, da Silva WJ, Del Bel Cury AA (2015). Evaluation of soidum hypochlorite as a denture cleanser: a clinical study. Gerodontology.

[16] Budtz-Jørgensen E (1979). Materials and methods for cleaning dentures. J Prosthet Dent.

[17] Baillie GS, Douglas LJ (1998). Effect of growth rate on resistance of *Candida albicans* biofilms to antifungal agents. Antimicrob Agents Chemother.

[18] Al-Huraishi H, Moran J, Jagger R, MacDonald E (2013). Evaluation of stain removal and inhibition properties of eight denture cleansers: an *in vitro* study. Gerodontology.

[19] Olsen HS, Falholt P (1998). The role of enzymes in modern detergency. J Surfactants Deterg.

[20] Rähse W (2014). Production of Tailor-made enzymes for detergents. Annu Rev Chem Biomol Eng.

[21] Hamza TA (2017). Bacterial protease enzyme: safe and good alternative for industrial and commercial use. I Int J Chem Biomol Sci.

[22] Gürkök S, editor. Microbial enzymes in detergents: a review. 4th international conference on advances in natural & applied sciences ICANAS; 2019.

[23] Saperas N, Fonfría-Subirós E (2011). Proteolytic enzymes in detergents: Evidence of their presence through activity measurements based on electrophoresis. J Chem Educ.

[24] Bryan PN (2000). Protein engineering of subtilisin. Biochim Biophys Acta.

[25] Walsh G (2007). Protein engineering: case studies of commercialized engineered products. Biochem Mol Biol Educ.

[26] Kwak J-Y, Lee D-H, Park Y-D, Kim S-B, Maeng J-S, Oh H-W (2006). Polyphasic assignment of a highly proteolytic bacterium isolated from a spider to *Serratia proteamaculans*. J Microbiol Biotechnol.

[27] Bersanetti PA, Park H-Y, Bae KS, Son K-H, Shin D-H, Hirata IY (2005). Characterization of arazyme, an exocellular metalloprotease isolated from *Serratia proteamaculans* culture medium. Enzyme Microb Technol.

[28] Lee K, Kim C-H, Kwon H-J, Kwak J, Shin D-H, Park D-S, Bae KS, Park H-Y (2004). Biochemical characterization of an extracellular protease in *Serratia proteamaculans* isolated form a spider. Korean J Microbiol.

[29] Mazorra-Manzano MA, Perea-Gutiérrez TC, Lugo-Sánchez ME, Ramirez-Suarez JC, Torres-Llanez MJ, González-Córdova AF (2013). Comparison of the milk-clotting properties of three plant extracts. Food Chem.

[30] Vijayakumar PP, Muriana PM (2015). A microplate growth inhibition assays for screening bacteriocins against *Listeria monocytogenes* to differentiate their mose-of-action. Biomolecules.

[31] Kwak J-Y, Lee K-E, Shin D-H, Maeng J-S, Park D-S, Oh H-W (2007). Biochemical and genetic characterization of arazyme, an extracellular metalloprotease produced from *Serratia proteamaculans* HY-3. J Microbiol Biotechnol.

[32] Kumar D, Savitri TN, Verma R, Bhalla TJRJM (2008). Microbial proteases and application as laundry detergent additive. Res J Microbiol.

[33] Hasan F, Shah AA, Javed S, Hameed AJA (2010). Enzymes used in detergents: lipases. Afr J Biotechnol..

[34] Beg QK, Gupta R (2003). Purification and characterization of an oxidation-stable, thiol-dependent serine alkaline protease from *Bacillus mojavensis*. Enzyme Microb Technol.

[35] Anwar A, Saleemuddin M (1997). Alkaline-pH-actingdigestive enzymes of the polyphagous insect pest *Spilosoma obliqua*: stabilityand potential as detergent additives. Biotechnol Appl Biochem.

[36] Banerjee UC, Sani RK, Azmi W, Soni R (1999). Thermostable alkaline protease from *Bacillus brevis* and its characterization as a laundry detergent additive. Process Biochem.

[37] Abidi F, Limam F, Nejib MM (2008). Production of alkaline proteases by *Botrytis cinerea* using economic raw materials: assay as biodetergent. Process Biochem.

[38] Rao CS, Sathish T, Ravichandra P, Prakasham RS (2009). Characterization of thermo-and detergent stable serine protease from isolated *Bacillus circulans* and evaluation of eco-friendly applications. Process Biochem.

[39] Wolff AL, Showell MS, Venegas MG, Barnett BL, Wertz WC, Bott R, Betzel C (1996). Laundry performance of subtilisin proteases. Subtilisin enzymes.

[40] Hmidet N, Ali NE-H, Haddar A, Kanoun S, Alya S-K, Nasri M (2009). Alkaline proteases and thermostable α-amylase co-produced by *Bacillus licheniformis* NH1: characterization and potential application as detergent additive. Biochem Eng J.

[41] Lima EMCX, Moura JS, Del Bel Cury AA, Garcia RCMR, Cury JA (2006). Effect of enzymatic and NaOCl treatments on acrylic roughness and on biofilm accumulation. J Oral Rehabil.

[42] Garcia RCMR, Júnior JADS, Rached RN, Del Bel Cury AA (2004). Effect of denture cleansers on the surface roughness and hardness of a microwave-cured acrylic resin and dental alloys. J Prosthodont.

[43] Budtz-Jörgensen E, Kelstrup J, Poulsen S (1983). Reduction of formulation of denture plaque by a protease (Alcalase®). Acta Odontol Scand.

[44] Kulak Y, Arikan A, Kazazoglu E (1997). Existence of *Candida albicans* and microorganisms in denture stomatitis patients. J Oral Rehabil.

[45] Ramage G, Saville SP, Thomas DP, Lopez-Ribot JL (2005). *Candida* biofilms: an update. Eukaryot Cell.

[46] Salerno C, Pascale M, Contaldo M, Esposito V, Busciolano M, Milillo L (2011). *Candida*-associated denture stomatitis. Med Oral Patol Oral Cir Bucal.

